# Optimisation of Simultaneous Tl-201/Tc-99m Dual Isotope Reconstruction with Monte-Carlo-Based Scatter Correction

**DOI:** 10.1155/2012/695632

**Published:** 2012-11-19

**Authors:** Tuija Kangasmaa, Jyrki Kuikka, Antti Sohlberg

**Affiliations:** ^1^Department of Radiation Therapy, Vaasa Central Hospital, Hietalahdenkatu 2-4, 65100 Vaasa, Finland; ^2^Department of Clinical Physiology and Nuclear Medicine, Kuopio University Hospital, P.O. Box 1777, 70211 Kuopio, Finland; ^3^Laboratory of Clinical Physiology and Nuclear Medicine, Joint Authority for Päijät-Häme Social and Health Care, Keskussairaalankatu 7, 15850 Lahti, Finland

## Abstract

Simultaneous Tl-201/Tc-99m dual isotope myocardial perfusion SPECT is seriously hampered by down-scatter from Tc-99m into the Tl-201 energy window. This paper presents and optimises the ordered-subsets-expectation-maximisation-(OS-EM-) based reconstruction algorithm, which corrects the down-scatter using an efficient Monte Carlo (MC) simulator. The algorithm starts by first reconstructing the Tc-99m image with attenuation, collimator response, and MC-based scatter correction. The reconstructed Tc-99m image is then used as an input for an efficient MC-based down-scatter simulation of Tc-99m photons into the Tl-201 window. This down-scatter estimate is finally used in the Tl-201 reconstruction to correct the crosstalk between the two isotopes. The mathematical 4D NCAT phantom and physical cardiac phantoms were used to optimise the number of OS-EM iterations where the scatter estimate is updated and the number of MC simulated photons. The results showed that two scatter update iterations and 10^5^ simulated photons are enough for the Tc-99m and Tl-201 reconstructions, whereas 10^6^ simulated photons are needed to generate good quality down-scatter estimates. With these parameters, the entire Tl-201/Tc-99m dual isotope reconstruction can be accomplished in less than 3 minutes.

## 1. Introduction

Tc-99m and Tl-201 are the two most commonly used isotopes in myocardial perfusion SPECT. Both isotopes have different benefits: Tc-99m has near ideal physical imaging properties for current gamma cameras, while Tl-201 acts more linearly according to the blood flow and allows the evaluation of myocardial viability. In order to take the full advantage of the properties of both isotopes, Tl-201/Tc-99m dual-isotope imaging has been suggested [1, 2]. Acquisition protocols where Tc-99m and Tl-201 are imaged separately have already been validated for clinical use [3, 4]. 

Simultaneous Tl-201/Tc-99m dual isotope acquisition has also gained some interest during the last couple of years. Simultaneous acquisition has several preferable properties compared to the separate protocol, namely perfect image registration between stress and rest images and faster patient throughput. This results in reduction in patient discomfort, and possible motion artefacts and production of identical physiological conditions during stress and rest scans. The main disadvantage of the simultaneous Tl-201/Tc-99m acquisition is the high down-scatter from Tc-99m into the Tl-201 window, which reduces the image quality of Tl-201-images and can interfere with the visualization of the possible perfusion defects. It has been shown that in order to use simultaneous Tl-201/Tc-99m acquisition protocol this cross-talk has to be corrected efficiently. Several correction methods have already been introduced with different results. However, there is currently no clear consensus with regards to how the down-scatter should be handled [5–11].

Monte-Carlo-(MC-) simulation-based scatter correction is one of the most general and accurate scatter correction methods available today [12]. MC-simulation is unfortunately very slow and heavy acceleration is needed in order to run reconstructions with MC-based scatter correction in clinically meaningful times. We have previously presented the reconstruction algorithm with MC-based scatter correction, which was accelerated using coarse grid and intermittent scatter modelling, allowing short reconstruction times [13].

The aim of this study was to extend our reconstruction method into dual isotope studies. Scatter correction in dual isotope studies is more demanding than in single isotope studies, due to the aforementioned Tc-99m to Tl-201 cross talk. Therefore the performance of the previously developed acceleration methods has to be validated for Tl-201/Tc-99m dual isotope myocardial perfusion SPECT. The goal was to optimise the new reconstruction algorithm in terms of reconstruction speed, without sacrificing image quality. 

## 2. Materials and Methods

### 2.1. Reconstruction Algorithm Description

Our previously published reconstruction algorithm is ordered subsets expectation maximisation (OS-EM) based. It uses rotation-based projectors and performs collimator modelling by incremental Gaussian diffusion and attenuation correction by multiplying ray-sums incrementally with appropriate attenuation coefficients and scatter correction using an efficient MC-simulator. The algorithm is described in more detail in [14] and it is used as the reconstruction engine of HERMES Medical Solutions' HybridRecon reconstruction package (Hermes Medical Solutions, Stockholm, Sweden). The algorithm was extended into Tl-201/Tc-99m dual isotope reconstruction by splitting the reconstruction into three steps: (1) Tc-99m reconstruction, (2) MC-based Tc-99m down-scatter simulation into the Tl-201 window, and (3) Tl-201 reconstruction incorporating the down-scatter estimate. The Tc-99m reconstruction is run using the previously published method and it should provide scatter-free Tc-99m isotope distribution. The reconstructed Tc-99m image is then used as an input for the same MC-simulator, which is used in the reconstruction for scatter-modelling and to simulate down-scatter from Tc-99m into the Tl-201 window. This down-scatter estimate is then added in the Tl-201 reconstruction to the Tl-201 self-scatter estimate.

The reconstruction speed is heavily influenced by the MC-simulator performance. We previously accelerated the MC-simulator by down-folding the MC-simulator input image to smaller matrix size (coarse grid modelling) and by reducing the number of OS-EM iterations where the scatter estimated is updated (intermittent scatter modelling). Both of these acceleration methods are based on the observation that scatter projections have relatively low resolution. Thus scatter can be calculated with bigger pixels and by updating scatter estimate only during the early OS-EM iterations where image resolution is lower.

In addition to reducing the matrix size and lowering the number of scatter update iterations MC simulation can also be accelerated by reducing the number of simulated photons. MC simulation is used in three different occasions in our dual isotope Tl-201/Tc-99m reconstruction method: during Tc-99m reconstruction, in Tc-99m down-scatter simulation, and during Tl-201 reconstruction. The number of simulated photons required has to be optimised for each occasion. The optimisation was performed using Monte Carlo simulated projection data of mathematical cardiac phantom and experimentally acquired projection data of a physical cardiac phantom. 

The number of OS-EM iterations and subsets, coarse factor, and post-filter parameters also affect the reconstruction. The number of iterations and subsets were chosen so that the contrast values did not increase with higher parameter values. Ten iterations and eight subsets were noticed to produce good quality data and subsequently used in all studies. The effect of coarse factor was not studied in detail, as it is used in an identical manner as in the previous study. Coarse factor was set to 2. All the reconstructed images were post-filtered with a 3D Gaussian post-filter whose full-width at half-maximum was 0.9 cm. This value was chosen by a visual evaluation of a large number of images. 

### 2.2. Phantom Studies

The 4-dimensional NURBS-based Cardiac-Torso (4D NCAT) phantom [15, 16] was used to optimise the reconstruction algorithm. Four female and four male phantoms were generated to simulate different outcomes of stress-Tl-201/rest-Tc-99m dual isotope studies, with reversible and irreversible defects and with a hot liver in the Tc-99m data.  Table 1 shows the summary of the phantom activity distributions [17] and defects. The attenuation map was simulated by using appropriate linear attenuation coefficients for lungs, soft tissue, and bone at Tl-201 and Tc-99m energies.

Projection data were generated using an MC-simulator developed by the authors [18]. SIEMENS Symbia SPECT/CT scanner (Siemens Healthcare, Erlangen, Germany) with Low Energy High Resolution (LEHR) collimators was simulated. Noise-free projection data of simultaneous dual isotope Tl-201/Tc-99m data and pure Tl-201 projection without Tc-99m down-scatter data were generated into 128 × 128 matrix size with 64 projections, 3.3 mm pixel size, and with energy window width of 15%. After simulation projection data were collapsed into 64 × 64 matrix size and Poisson-noise was added the number of total counts in the Tl-201 window in the dual isotope study was 80 Mcts, 76 Mcts in the Tc-99m window, and 10 Mcts in the pure Tl-201 study.

The Jaszczak phantom with a cardiac insert with fillable defects (Data Spectrum Corporation, Hillsborough, NC, USA) was used to verify the simulation study results.  Table 2 shows cardiac insert and defect activities. The defects were set in the anterior and inferior walls, with the defect in the anterior wall being reversible and the defect in the inferior wall irreversible. 

Phantom data were acquired with SIEMENS Symbia SPECT/CT scanner with LEHR collimators. The dual isotope acquisition was performed with 64 × 64 matrix size, 64 projections, two 15% energy windows centered on 72 keV and 140 keV, and 25 s acquisition time per projection. Accompanying CT was acquired with 512 × 512 matrix, 0.98 mm pixel size, 130 keV voltage and 17 mAs, and 1.5 pitch. CT was later converted into attenuation map using bilinear scaling. Pure Tl-201 data were acquired using the same filled phantom as was used in the dual isotope acquisition, but the acquisition was performed 72 hours after the dual isotope study, when Tc-99m activity had nearly completely decayed. The acquisition protocol for the pure Tl-201 acquisition was almost equal to the dual isotope protocol. The only difference was the longer 40 s projection time, which compensated the decay in Tl-201 activity.

### 2.3. Reconstruction Algorithm Optimisation

The reconstruction algorithm performance was optimised in three phases using the Monte Carlo simulated projection data described above. Ten scatter update iterations and 10^6^ simulated photons in both the Tc-99m and Tl-201 reconstructions and 10^7^ down-scatter simulated photons were used as a starting point and reference level for the optimisation. The optimisation was initiated by studying the effect of scatter update iterations and number of simulated photons in the Tc-99m reconstruction. 

#### 2.3.1. Tc-99m Reconstruction Optimisation

**Figure 1:**
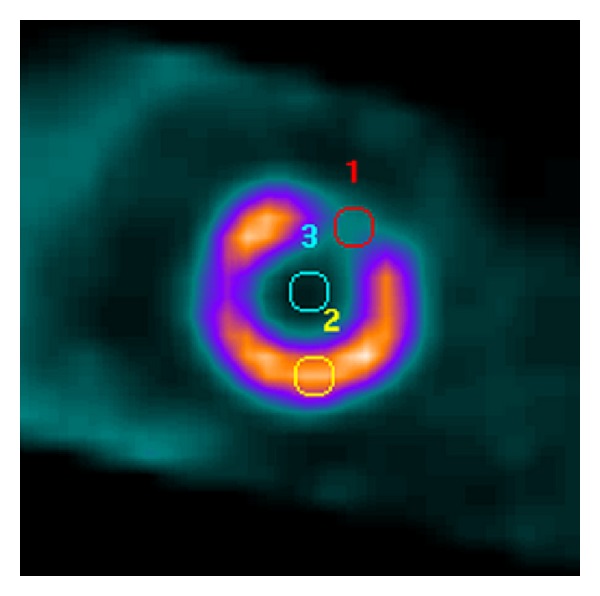
Example of ROI positions that were used for contrast analyses. ROI number 1 is on the defect, number 2 is on the healthy myocardium, and number 3 is on LV.

The Tc-99m reconstruction was optimised by comparing 2 and 10 scatter update iterations with 10^5^ and 10^6^ simulated photons while keeping the number of down-scatter simulated photons at 10^6^. The defect-to-healthy myocardium and left-ventricle-(LV-) to-healthy myocardium contrasts were used to compare the different parameters. The contrasts were calculated as:

(1) $$\begin{array}{c} {\text{contrast}}_{\text{myocardium\_defect}}=\frac{C_{\text{myocardium}}-C_{\text{defect}}}{C_{\text{myocardium}}},\\ {\text{contrast}}_{\text{myocardium\_LV}}=\frac{C_{\text{myocardium}}-C_{\text{LV}}}{C_{\text{myocardium}}}, \end{array}$$

where *C* corresponds to the total counts of the region mentioned in the subscript. The counts were obtained by drawing regions of interest (ROI) with same area on the defect, myocardium, and LV on the oblique plane where the defect was best visible.  Figure 1 shows an example of the positioning of the ROIs. 

**Figure 2:**
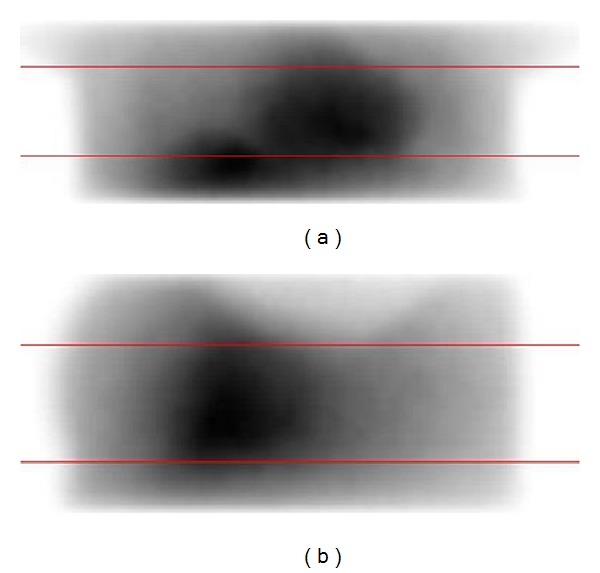
Profiles through anterior (a) and lateral (b) down-scatter projections. The measured profile width was 10 pixels.

In addition to Tc-99m image contrast, the down-scatter projection images were also analysed by plotting profiles through the anterior and lateral down-scatter projections (Figure 2). 

#### 2.3.2. Down-Scatter Simulation Optimisation

After Tc-99m reconstruction optimisation the effect of the number of down-scatter simulated photons was studied. Down-scatter was simulated with 10^5^, 10^6^, and 10^7^ photons and Tl-201 reconstruction was run using each down-scatter estimate with 10 scatter update iterations and 10^6^ Tl-201 scatter photons. The reconstructed Tl-201 images were analysed by investigating Tl-201 image contrasts and down-scatter projection profiles in a similar manner as in the Tc-99m reconstruction optimisation. 

#### 2.3.3. Tl-201 Reconstruction Optimisation

The final step in the optimisation process was to study the effect of scatter update iterations and the number of simulated photons in the Tl-201 reconstruction. The previously optimised Tc-99m reconstruction and down-scatter simulation parameters were used for the two first steps of the reconstruction. Tl-201 reconstruction was optimised by comparing 2 and 10 scatter update iterations and with 10^5^ and 10^6^ simulated photons. The reconstructed Tl-201 image contrasts were analysed as in the previous steps.

The Jaszczak phantom dual isotope study was reconstructed using the parameters optimised with the simulated data. These images were compared with images obtained using completely unoptimised reconstruction parameters and with reconstructed images obtained using pure Tl-201 data. The contrast analysis for the reconstructed Tl-201 data was performed as in the previous steps. The Tc-99m images were not studied.

## 3. Results

### 3.1. Tc-99m Reconstruction Optimisation

**Figure 3:**
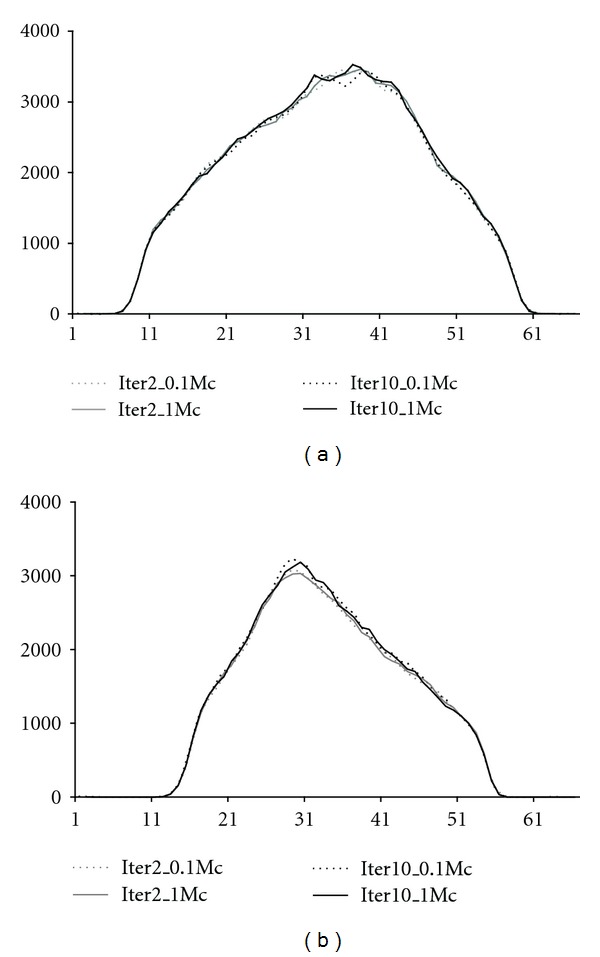
Profiles through anterior (a) and lateral (b) down-scatter projections. The profiles were obtained with 2 and 10 scatter update iterations (iter2 and iter10) and 10^5^ (0.1 Mc) and 10^6^ (1.0 Mc) simulated photons in the Tc-99m reconstruction.

Table 3 and Figure 3 show the Tc-99m reconstruction optimisation results for the contrast and profile analyses, respectively, for one phantom only. These results suggest that in Tc-99m reconstruction 2 scatter update iterations and 10^5^ simulated photons produce nearly identical images compared to reconstruction with 10 scatter update iterations and 10^6^ simulated photons.

### 3.2. Down-Scatter Simulation Optimisation

**Figure 4:**
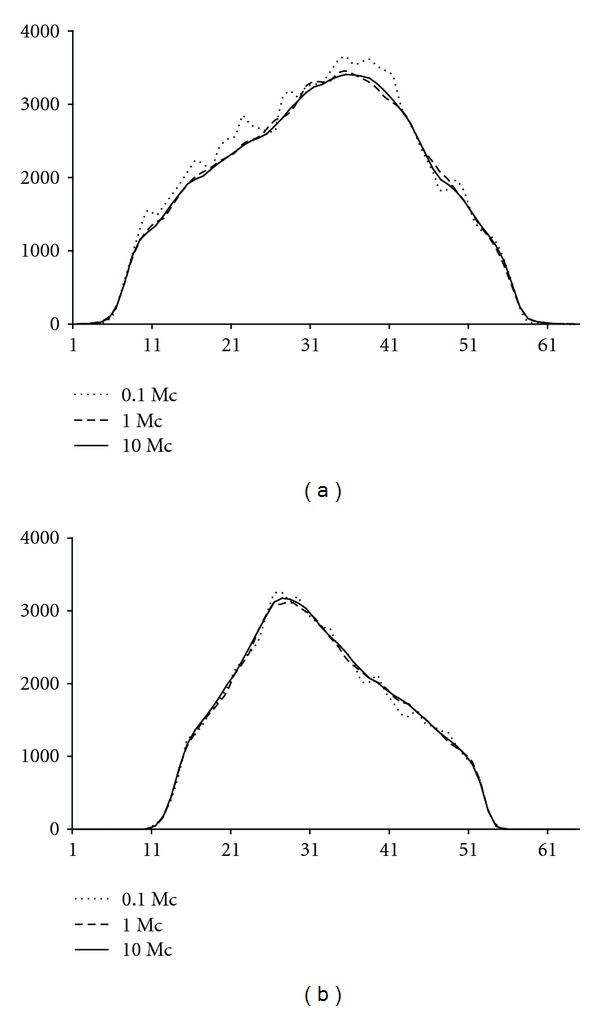
Profiles through anterior (a) and lateral (b) down-scatter projections. The profiles were obtained with 10^5^ (0.1 Mc), 10^6^ (1.0 Mc), and 10^7^ (10 Mc) simulated down-scatter photons.

Table 4 and Figure 4 present the down-scatter simulation optimisation results for the contrast and profile analyses, respectively, for one phantom only. The results show that 10^6^ simulated down-scatter photons provide contrasts and down-scatter projections that are nearly identical to 10^7^ simulated down-scatter photons. With 10^5^ simulated down-scatter photons the profiles are too noisy.

### 3.3. Tl-201 Reconstruction Optimisation

Table 5 shows the Tl-201 reconstruction optimisation results obtained with one phantom only. The table shows that 2 scatter update iterations and 10^5^ simulated photons are enough for the Tl-201 reconstruction.

According to the results shown above, the optimised parameters for the Tl-201/Tc-99m dual isotope reconstruction are 2 scatter update iterations and 10^5^ simulated photons for the Tc-99m and Tl-201 reconstruction and 10^6^ simulated down-scatter photons. 

**Figure 5:**
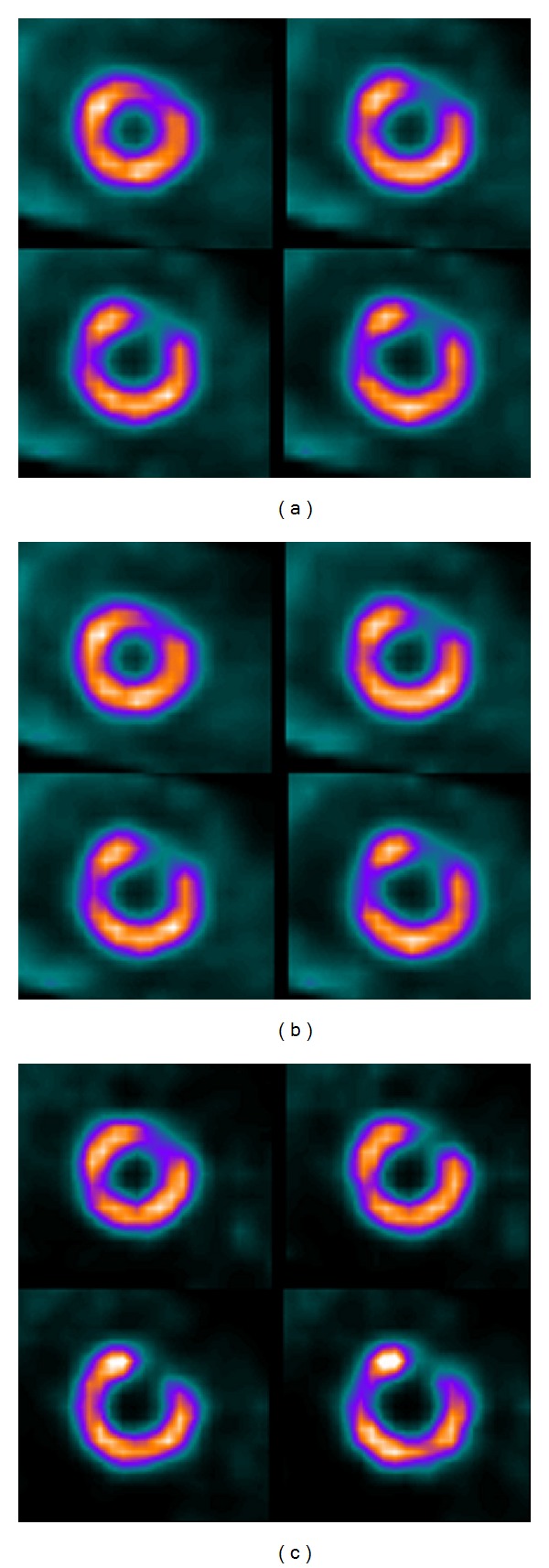
Example Tl-201 short-axis slices of the NCAT phantoms. Optimised Tc-99m/Tl-201 reconstruction result is shown in (a), unoptimised in (b), and reconstruction from pure Tl-201 data in (c).

These optimised parameters were further used to compare optimised and un-optimised reconstructions (10 scatter update iterations and 10^6^ simulated photons for the Tc-99m and Tl-201 reconstruction and 10^7^ simulated down-scatter photons) to reconstructions from pure Tl-201 data using all the simulated phantoms. Pure Tl-201 data were reconstructed using 2 scatter update iterations and 10^5^ simulated photons.  Table 6 and Figure 5 present these results. The results show that optimised parameters provide contrast values that are equal with the results obtained with un-optimised parameters, but that the reconstruction times are much shorter. The down-scatter compensation cannot, however, fully correct for the down-scatter as can be seen by comparing the contrast values and the images of the dual-isotope and pure Tl-201 reconstructions.

**Figure 6:**
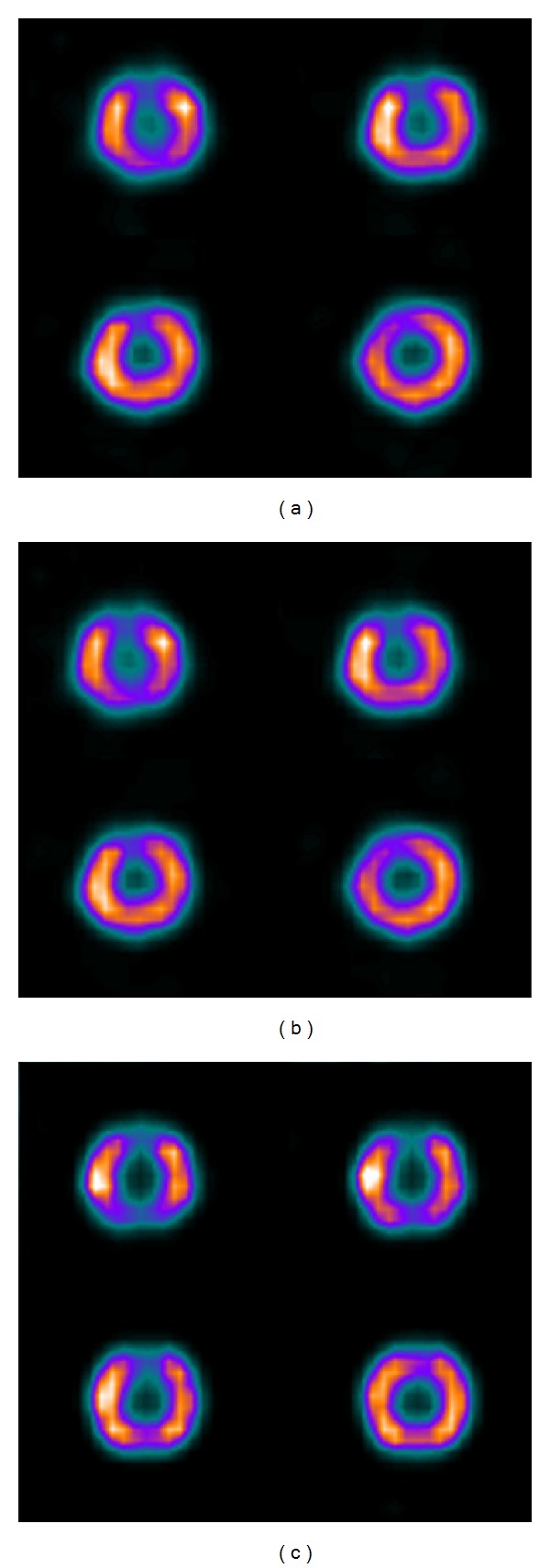
Example Tl-201 short-axis slices of the Jaszczak phantom with the cardiac insert. Optimised Tc-99m/Tl-201 reconstruction result is shown in (a), unoptimised in (b), and reconstruction from pure Tl-201 data in (c).

Table 7 and Figure 6 compare the optimised and un-optimised dual isotope reconstruction with reconstruction obtained with pure Tl-201 data of the Jaszczak phantom with the cardiac insert. These physical phantom study results confirm the simulation study findings: optimised parameters give equal contrasts when compared to un-optimised reconstruction, but down-scatter correction cannot fully reach the level of pure Tl-201 data results.

## 4. Discussion

In this study our previously presented reconstruction algorithm was extended and optimised for dual isotope Tl-201/Tc-99m studies in terms of reconstruction speed. The new dual isotope algorithm performs Tl-201/Tc-99m reconstructions by splitting the reconstruction into Tc-99m reconstruction, Tc-99m down-scatter simulation, and Tl-201 reconstruction incorporating the down-scatter estimate. Two scatter update iterations and 10^5^ simulated photons for the Tc-99m and Tl-201 reconstructions and 10^6^ simulated down-scatter photons were found to provide accurate results in clinically acceptable reconstruction times (Table 6).

The 2 scatter update iterations and 10^5^ have been found to suffice also for single isotope Tc-99m reconstruction [14, 19]. Simulating more scatter photons does not lead to improvement in either ventricular or lesion contrast. For the down-scatter estimation, however, we used more photons in order to make the down-scatter estimate less noisy. The contrast values in the down-scatter compensated Tl-201 images are practically identical for 10^5^, 10^6^, and 10^7^ simulated down-scatter photons (Table 4) but 10^5^ photons seems to generate much noisier down-scatter estimate than 10^6^ photons. This noise might progress to the reconstructed images if the Tl-201 activity is very low.

The developed down-scatter correction method cannot fully compensate for the Tc-99m/Tl-201 cross-talk as can be seen by comparing the results obtained with the dual isotope data and pure Tl-201 data in Tables 6 and 7. The pure Tl-201 data contrasts are better than the down-scatter compensation contrasts. This can be explained by the lead X-ray emissions, which occur when Tc-99m photons hit the collimator. These X-rays are emitted at the Tl-201 energy level and they contaminate the Tl-201 data. Currently our reconstruction method corrects only for patient scatter, but more accurate correction might be needed. De Jong et al. [11] have included lead X-ray correction into their Tl-201/Tc-99m reconstruction method and have shown that it improves image quality. The only problem in including collimator effects into MC-based scatter correction is the increase in reconstruction time. The common Gaussian collimator model, which was also used in this work, is very efficient. Changing that to something more complicated will definitely have a big effect on the reconstruction times and it will also make reconstruction algorithm implementation more challenging.

Our reconstruction method has similar features to the methods published by Kadrmas et al. [10] and de Jong et al. [11]. Both of these methods also broke the reconstruction into three parts: Tc-99m reconstruction, Tc-99m down-scatter simulation, and Tl-201 reconstruction. Kadrmas et al. used effective source scatter estimation for scatter modelling, whereas de Jong et al. used Monte Carlo as we did. We focused also on optimising the reconstruction parameters, which is very important for an algorithm to be clinically acceptable. In our case the reconstruction parameter optimisation reduced the reconstruction times to approximately 1/4 of the un-optimised reconstruction times (Table 6). At the moment our dual isotope reconstruction algorithm is limited to Tl-201/Tc-99m reconstructions. The same concept could, however, probably be extended to other isotope pairs or to isotopes with several peaks. Reconstruction would always start with the isotope or energy peak, which has the highest energy. This data would be reconstructed and downscattered to lower windows and subsequent reconstructions would run until the isotope or energy peak with the lowest energy is reached. 

Dual isotope imaging with simultaneous acquisition is clinically attractive, as it increases the possible patient throughput and reduces patient discomfort. Dual isotope imaging also offers perfect alignment and identical physiological conditions between stress and rest images, which may give additional information to the physician.

This study has limitations. MC-simulated projection data and physical phantoms were used instead of real patient studies. We tried to compensate the lack of real patient data by using realistic phantoms and we also tried to adjust the activity levels to clinically meaningful values. We believe that our optimised reconstruction method works well also with patient data but a large number of patient studies are still required to validate our method and the entire Tl-201/Tc-99m dual isotope SPECT. 

## 5. Conclusion

The newly developed Tl-201/Tc-99m reconstruction algorithm was efficiently accelerated using a reduced number of scatter update iterations and simulated photons. Two scatter update iterations and 10^5^ simulated photons for the Tc-99m and Tl-201 reconstructions and 10^6^ simulated down-scatter photons were sufficient for good quality images.

## Figures and Tables

**Table 1:** The activity configuration of the NCAT phantoms. The table shows the relative activity concentrations per pixel and the defect parameters for the NCAT phantoms. The same parameters were used for both the female and male phantoms

Phantom	Isotope	Myocardium activity	Ventricle activity	Lung activity	Liver activity	Back-ground tissue activity	Defect position	Activity in defect versus myocardium (%)
1	Tl-201	50	3	2	3	3	ANT SEPT	20 40
	Tc-99m	180	6	18	90	6	ANT SEPT	20 40

2	Tl-201	50	3	2	3	3	ANT INF	40 20
	Tc-99m	95	3	9	189	3	—	—

3	Tl-201	50	3	2	3	3	ANT SEPT	20 40
	Tc-99m	180	6	18	90	6	ANT	20

4	Tl-201	50	3	2	3	3	ANT INF	40 20
	Tc-99m	95	3	9	189	3	INF	20

**Table 2:** The activity configuration for the physical phantom. The table shows cardiac insert and defect activities used for the physical phantom study.

Isotope	Myocardium activity (MBq)	Ventricle activity (MBq)	Defect activity (MBq)	Defect position
Tl-201	8.18	0.34	0.04 0.04	ANT INF
Tc-99m	35.96	1.25	0.17	INF

**Table 3:** The contrast values for the Tc-99m optimization. In this table myocardium versus defect and myocardium versus left ventricle (LV) contrasts are presented for the Tc-99m optimisation. The contrast values were calculated for Phantom 3 that had an anterior defect.

Number of simulated photons in Tc-99m reconstruction	Number of down-scatter simulated photons	Number of scatter update iterations	Myocardium versus defect contrast	Myocardium versus LV contrast
10^5^	10^6^	2	**0.74**	**0.97**
10^5^	10^6^	10	**0.73**	**0.97**
10^6^	10^6^	2	**0.73**	**0.97**
10^6^	10^6^	10	**0.73**	**0.97**

**Table 4:** The contrast values for the down-scatter optimisation. In this table myocardium versus defect and myocardium versus left ventricle (LV) contrasts are presented for the down-scatter optimisation. The contrast values were calculated for Phantom 3 for the anterior defect. The Tc-99m image that served as an input for the down-scatter simulation was reconstructed using the parameters optimised in the previous step.

Number of simulated photons in Tl-201 reconstruction	Number of down-scatter simulated photons	Number of scatter update iterations in Tl-201 reconstruction	Myocardium versus defect contrast	Myocardium versus LV contrast
10^6^	10^5^	10	**0.67**	**0.90**
10^6^	10^6^	10	**0.67**	**0.89**
10^6^	10^7^	10	**0.68**	**0.89**

**Table 5:** The contrast values for the Tl-201 optimization. In this table myocardium versus defect and myocardium versus left ventricle (LV) contrasts are presented for the Tl-201 reconstruction optimisation. The contrast values are calculated for Phantom 3 for the anterior defect.

Number of simulated photons	Number of down-scatter simulated photons	Number of scatter update iterations	Myocardium versus defect contrast	Myocardium versus LV contrast
10^5^	10^6^	2	**0.68**	**0.89**
10^5^	10^6^	10	**0.67**	**0.89**
10^6^	10^6^	2	**0.67**	**0.89**
10^6^	10^6^	10	**0.68**	**0.89**

**Table 6:** Contrast evaluation with NCAT phantoms. The table shows myocardium versus defect and myocardium versus left ventricle (LV) contrasts for optimised and unoptimised Tl-201/Tc-99m reconstruction and for pure Tl-201 reconstruction. The results were obtained using all the Monte Carlo simulated NCAT phantoms, and the contrasts are average values of the corresponding female and male phantoms. Only Tl-201 reconstruction results are shown.

Defect	Reconstruction	Myocardium versus defect contrast	Myocardium versus LV contrast	Reconstruction time (min)
ANT	Optimised	**0.62**	**0.91**	**<3**
	Unoptimised	**0.61**	**0.91**	** <13**
	Pure Tl-201	**0.71**	**0.96**	**<1**

INF	Optimised	**0.80**	**0.94**	**<3**
	Unoptimised	**0.81**	**0.94**	** <13**
	Pure Tl-201	**0.90**	**0.97**	**<1**

SEPT	Optimised	**0.70**	**0.96**	**<3**
	Unoptimised	**0.67**	**0.95**	** <13**
	Pure Tl-201	**0.64**	**0.99**	**<1**

**Table 7:** Contrast evaluation with the physical phantom. The table shows myocardium versus defect and myocardium versus left ventricle (LV) contrasts for optimised and unoptimised Tl-201/Tc-99m reconstruction and for pure Tl-201 reconstruction. The results were obtained using the Jaszczak phantom with a cardiac insert. Only Tl-201 reconstruction results are shown.

Defect	Reconstruction	Myocardium versus defect contrast	Myocardium versus LV contrast
ANT	Optimised	**0.60**	**0.75**
	Unoptimised	**0.59**	**0.75**
	Pure Tl-201	**0.72**	**0.87**

INF	Optimised	**0.46**	**0.63**
	Unoptimised	**0.48**	**0.63**
	Pure Tl-201	**0.65**	**0.86**
